# A *CNGB1* Frameshift Mutation in Papillon and Phalène Dogs with Progressive Retinal Atrophy

**DOI:** 10.1371/journal.pone.0072122

**Published:** 2013-08-28

**Authors:** Saija J. Ahonen, Meharji Arumilli, Hannes Lohi

**Affiliations:** 1 Department of Veterinary Biosciences and Research Programs Unit, Molecular Neurology, University of Helsinki, Helsinki, Finland; 2 The Folkhälsan Institute of Genetics, Helsinki, Finland; University of Texas MD Anderson Cancer Center, United States of America

## Abstract

Progressive retinal degenerations are the most common causes of complete blindness both in human and in dogs. Canine progressive retinal atrophy (PRA) or degeneration resembles human retinitis pigmentosa (RP) and is characterized by a progressive loss of rod photoreceptor cells followed by a loss of cone function. The primary clinical signs are detected as vision impairment in a dim light. Although several genes have been associated with PRAs, there are still PRAs of unknown genetic cause in many breeds, including Papillons and Phalènes. We have performed a genome wide association and linkage studies in cohort of 6 affected Papillons and Phalènes and 14 healthy control dogs to map a novel PRA locus on canine chromosome 2, with a 1.9 Mb shared homozygous region in the affected dogs. Parallel exome sequencing of a trio identified an indel mutation, including a 1-bp deletion, followed by a 6-bp insertion in the *CNGB1* gene. This mutation causes a frameshift and premature stop codon leading to probable nonsense mediated decay (NMD) of the *CNGB1* mRNA. The mutation segregated with the disease and was confirmed in a larger cohort of 145 Papillons and Phalènes (P_Fisher_ = 1.4×10^−8^) with a carrier frequency of 17.2 %. This breed specific mutation was not present in 334 healthy dogs from 10 other breeds or 121 PRA affected dogs from 44 other breeds. CNGB1 is important for the photoreceptor cell function its defects have been previously associated with retinal degeneration in both human and mouse. Our study indicates that a frameshift mutation in *CNGB1* is a cause of PRA in Papillons and Phalènes and establishes the breed as a large functional animal model for further characterization of retinal *CNGB1* biology and possible retinal gene therapy trials. This study enables also the development of a genetic test for breeding purposes.

## Introduction

Progressive retinal degeneration or atrophy is one of the most common causes of blindness both in human and in dog affecting the retinal photoreceptor cells. The most typical type of canine progressive retinal atrophy (PRA) is primary rod degeneration, where the light sensitive rod photoreceptor cells degenerate, resulting visual impairment in the dark light. When the disease progresses to the cone cells, which function in the day light, the vision is completely lost [1]. Ophthalmoscopically the initial clinical signs include tapetal hyperreflectivity, retinal vascular attenuation and pigment changes and atrophy of the optic nerve. [2]

Various forms of PRA have been diagnosed in different breeds of dog, some breeds have been found to manifest more than one type of the disease. Most canine PRAs are recessively inherited and 12 causative genes are known including *ADAM metallopeptidase domain 9* (*ADAM9*) [3], *bestrophin* (*BEST1*) [4], *chromosome 2 open reading frame 71* (*C2orf71*) [5], *cyclic nucleotide gated channel beta 3* (*CNGB3*) [6], *nephronophthisis* (*NPHP4*) [7], *phosphodiesterase 6A, cGMP-specific, rod, alpha* (*PDE6A*) [8], *rod cGMP-specific 3',5'-cyclic phosphodiesterase subunit beta* (*PDE6B*) [9], *rhodopsin* (*RHO*) [10], *retinitis pigmentosa GTPase regulator-interacting protein* (*RPGRIP1*) [11], *retinal pigment epithelium-specific protein* (*RPE65*) [12], *solute carrier family 4, anion exchanger, member 3* (*SLC4A3*) [13], *progressive rod-cone degeneration* (*PRCD*) gene [14]. Furthermore, three X-linked canine PRAs are known [15], [16]. However, there are still other PRA affected breeds of unknown genetic cause.

Papillon breed is affected with an autosomal recessive late onset PRA with a mean onset at 5.6 years of age [17]. Based on the electroretinogram (ERG) recordings, affected dogs have a primary loss of the rod photoreceptor cells, followed by loss of cone cell function [18], [19]. The first clinical signs are seen as difficulties in the dim light. The disease progress very slowly and the affected dogs seem to be visually normal throughout their life, as the cone function is fairly well preserved [18], [19]. The ophthalmoscopical signs include increased tapetal reflectivity and retinal vascular attenuation followed by pigment migration in the non-tapetal fundus [17].

According to Fèdèration Cynologique Internationale (FCI), Papillon breed is separated to two different breeds, Papillon and Phalène in Europe. Separation is based on the ear morphology, Papillons having bricked ears and Phalènes more low set ears. Genetic background is expected to be similar as both types of dogs are born in the same litter and puppies are registered in Europe based on their ear morphology. The American Kennel Club registers both types as Papillons. Due to shared breed origin, it is reasonable to hypothesize that the genetic cause of PRA is shared.

We have established a pedigree and a study cohort of PRA affected and unaffected Papillons and Phalènes to identify the cause of the disease. We performed a genome wide association, linkage and exome sequencing studies to map the disease to a region in CFA2 and to identify a frameshift-causing indel mutation in the *cyclic nucleotide gated channel beta 1* (*CNGB1*) gene in the affected dogs.

## Materials and Methods

### Study cohort

Blood and buccal swab samples were collected from pet dogs to the Dog DNA bank at the University of Helsinki, Finland. All samples were submitted by the owners’ consent and were collected under the permission of animal ethical committee of County Administrative Board of Southern Finland (ESAVI/6054/04.10.03/2012). Genomic DNA was extracted from EDTA blood samples, using Chemagic Magnetic Separation Module I (MSM I) (Chemagen Biopolymer-Technologie AG, Baeswieler, Germany) according to the manufacturer’s instructions. DNA from buccal swabs (Eurotubo Cytobrush, sterile, 200mm, Danlab, Helsinki, Finland) was extracted using QIAamp DNA Mini Kit (Qiagen). A retinal sample from an Australian Cattle Dog, euthanized for unrelated causes and free of PRA, was taken post mortem with owner’s consent. RNA was extracted using RNeasy Mini Kit (Qiagen) according to manufacturer’s instructions. DNA and RNA concentrations were measured using Nanodrop ND-1000 UV/Vis Spectrophotometer (Nanodrop technologies, Wilmington, Delaware, USA) and stored at -20°C (DNA) or −80°C (RNA).

We have collected altogether samples from 59 Papillons and from 116 Phalènes in our Dog DNA bank. We selected clinically confirmed cases for our genetic studies. Samples were collected from affected Papillons (n = 4) or Phalènes (n = 2) with clinical signs consistent with PRA, including tapetal hyper-reflectivity, retinal pigmentary changes and vascular attenuation. Control dogs (n = 14) in the mapping study were eye examined healthy over seven years of age.

A pedigree was constructed around the affected dogs using the genealogical data available in public canine registries such as the Finnish Kennel Club’s Koiranet, the Swedish Kennel Club’s Hunddata databases or as informed by the owners. A GenoPro genealogy software was utilized to draw the pedigree.

### Genome wide association study

A genome-wide association study (GWAS) was performed using Illumina’s CanineHD BeadChip arrays (San Diego, CA, USA) for 6 cases (4 Papillons, 2 Phalènes) and 14 controls (3 Papillons, 11 Phalènes). Genotyping was performed in our core facility at the FIMM Technology Center. Quality control procedures were included when analyzing the data. Only SNPs which met Hardy-Weinberg expectations P≤0.0001, had ≥95% genotyping rate and minor allele frequency (MAF) of 5% were included in the analysis, resulting in the exclusion of 64640 SNPs out of 173662. The GWAS data is available from the researchers upon request.

To compare the allele frequencies, a case-control association test was performed using PLINK 1.07 [20]. Significance values from these analyses were used to generate a whole-genome association plot using the statistical package R [21]. Identity-by-state (IBS) clustering and CMH meta-analysis (PLINK) were used to adjust for population stratification. Genome-wide corrected empirical p-values were determined by applying 100.000 permutations to the data. The GWAS data was also re-analyzed (PLINK) by adjusting for the strongest SNP on CFA2 (BICF2P309315) to detect the presence of any modifier loci.

Besides association, the genotyping data was analyzed using a joint family-based linkage and association analysis program Pseudomarker [22]. The family-based analyses were performed under a recessive inheritance model, and included parametric single-point linkage test, association analysis (LD|Linkage) and joint analysis (LD+Linkage).

### Exome sequencing

**Figure 1:**
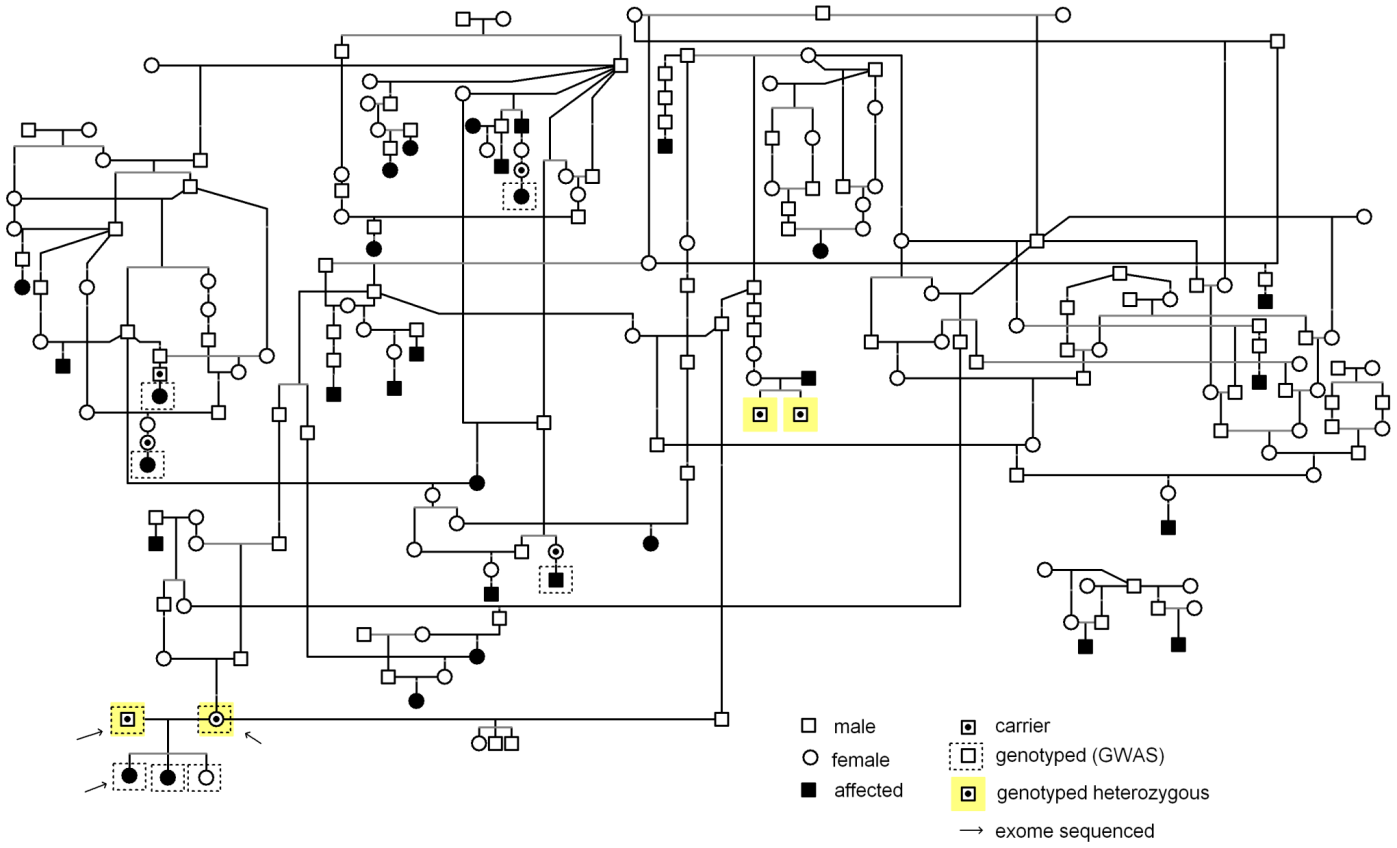
Pedigree of PRA affected Papillons and Phalènes. Pedigree indicates the affected dogs that were used in the study. Samples from six affected dogs were available for genotyping. Disease segregation is consistent with autosomal recessive mode of inheritance as all affected dogs are born from healthy parents and both sexes are affected. Obligate carrier parents of affected dogs are marked in the pedigree. Obligate carriers genotyped as heterozygous for *CNGB1* mutation are marked with a yellow background.

Exome sequencing was performed for a trio of Phalènes, including an affected proband and healthy parent dogs (Fig. 1). The coding sequences were captured using Agilent’s Canine Exome Capture Kit (Agilent, Santa Clara, CA, USA) that was designed based on the build 2.1 of the canine genome reference sequence. The capture included a 54 Mb design that covered the Ensemble and RefSeq Genes from the UCSC track, human protein alignments and spliced ESTs that lie outside of Ensemble. The capture was performed according the manufacturer’s instruction and the sequencing was performed using the Illumina HiSeq2000. Both the capture and sequencing were performed at the FIMM technology center. The exome sequencing data is available upon request.

The data analysis included quality control, alignment, variant calling and annotation of the variants. Quality control was performed using FASTX toolkit (http://hannonlab.cshl.edu/fastx_toolkit/index.html) to remove the low quality raw-data and to reduce the false positives, followed by alignment of the reads to the build 2.1 of the canine genome reference sequence with Burrows-Wheeler (BWA) aligner tool [23]. The aligned reads were then directed to the variant calling programs GATK [24] and Samtools [25] to identify the SNPs and short indels present within the exome samples. Pindel [26] program was used to identify the structural variants. Finally, the identified variants were annotated against NCBI and Ensembl databases to find out the variants present in the coding and non-coding regions and to the dbSNP131 to identify known polymorphic variants. The annotation was performed using ANNOVAR [27] and in-house custom R-scripts. The data was then filtered assuming a recessive mode of inheritance using in-house R-scripts.

### Mutation screening and validation

An indel in exon 25, including 1-bp deletion followed by 6-bp insertion was found in the *cyclic nucleotide gated channel beta 1* (*CNGB1*) and a non-synonymous variant in *2-oxoglutarate and iron-dependent oxygenase domain containing 1* (*OGFOD1*) genes. These variants were confirmed by PCR in additional cases and controls. PCR primer pairs were designed using Primer3 [28] software to amplify the variants from genomic DNA. The mutation on exon 25 of the *CNGB1* gene was amplified from 6 PRA affected and 14 control Papillons and Phalènes and one Rough Collie using forward primer 5'-AACAATCTCCTGGAGCCTCA-3' and reverse primer 5'-TGGAAGCAACGGTAGAGAAGA-3'. The non-synonymous mutation on exon 9 of the *OGFOD1* gene was amplified from 6 PRA affected and 10 controls dogs and one Rough Collie with forward primer 5'-CTTGTGCTTGAGTTGGATGTG-3' and reverse primer 5'-TGGGAGAGCGAAGACTTTGT-3'.

The best associated SNP on CFA1 (BICF2P839236) was amplified in a larger sample cohort of 6 affected and 168 controls using forward primer 5'-TCCAATCCTTAATGTTCTAAAGTGAA-3' and reverse primer 5'-CCCAGATTAAGATGATTCACCA-3'.

To further investigate the disease associated variant in a larger sample cohort the *CNGB1* variant was sequenced from 43 Papillons and 93 Phalènes. In addition, the mutation was studied in 121 dogs from other breeds affected with PRA or retinal degeneration, representing 44 breeds (Table S3) and 334 healthy control dogs from 10 breeds, including Australian Shepherd (n = 20), Border Collie (n = 92), Chihuahua (n = 19), Finnish Lapphund (n = 93), Japanese Chin (n = 14), Shetland Sheepdog (n = 20), Silk Terrier (n = 20), Toy Poodle (n = 20), West Highland White Terrier (n = 20) and Wire Haired Dachshund (n = 16).

The PCR reactions were carried in 12 μl reactions consisting of 0.6 U Biotools polymerase (Biotools, Madrid, Spain), 200 μM dNTPs (Thermo Scientific), 1.5 mM MgCl_2_ (Biotools), 1 x PCR buffer (Biotools), 0.83μl of forward and reverse primers (Sigma Aldrich) and 10 ng template genomic DNA or mRNA. The reaction mixtures were subjected to a thermal cycling program of 95°C for 10 min, followed by 35 cycles of 95°C for 30 s, 30 s at the annealing temperature 60°C, and 72°C for 60 s and final elongation stage of 72°C for 10 min. The PCR products were purified using ExoSap (Thermo Scientific) according to manufacturer’s instructions and sequenced using the Sanger sequencing. Sequence analysis was performed using Sequencher software (Gene Codes, Ann Arnbor, MI, USA).

The *CNGB1* mRNA sequence (XM_848817.1) was amplified using Biotools (Biotools) polymerase and PCR protocol described above with annealing temperature of 62°C. The primers used for amplification are listed in supplementary data (Table S1).

## Results

We established a cohort of PRA affected and unaffected Papillons and Phalènes to map the disease locus and to identify the genetic cause. Clinical signs reported from the affected dogs were consistent with PRA, including tapetal hyper-reflectivity and vascular attenuation. The average age of the PRA diagnosis was 4.3 years. The pedigree established around the affected dogs suggested a recessive mode of inheritance (Fig. 1).

**Figure 2:**
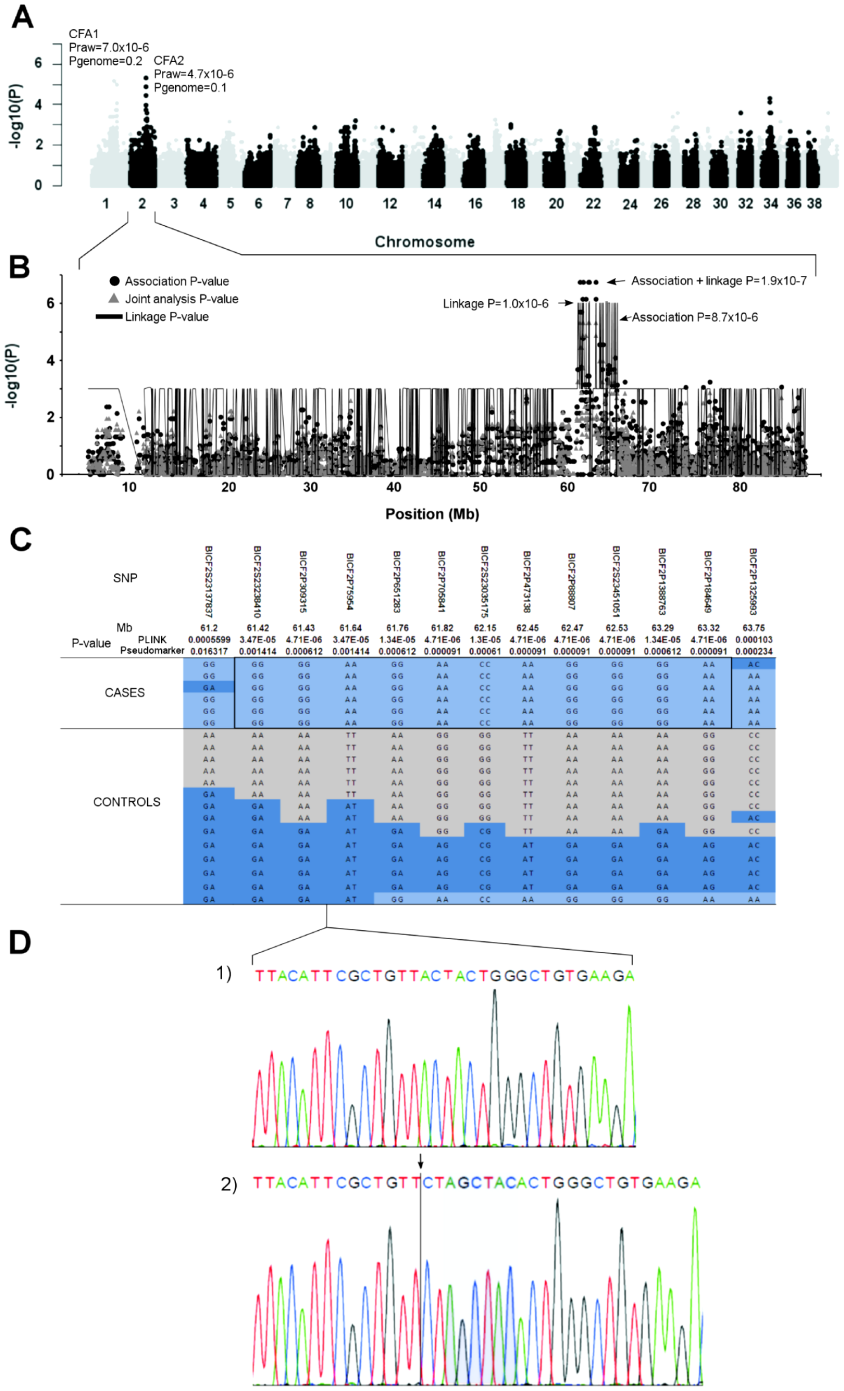
Genome wide association and linkage analyses. **A**) A Manhattan plot of genome-wide case-control association analysis performed using 6 cases and 14 controls indicate the most highly associated region in CFA2. **B**) The PRA associated region on chromosome 2 spans from 61.4 Mb to 63.3 Mb based on association, linkage and joint analyses. **C**) Genotypes at the PRA associated region on CFA2. All cases share a 1.9 Mb homozygous block, and within this block SNPs BICF2S23238410, BICF2P309315 and BICF2P75954 show complete recessive segregation with the disease. **D**) Chromatograms of the c.2685delA (arrow), c.2687_2688insTAGCTA (shadowed) mutations in *CNGB1* gene in an affected (2) and normal (1) dog. The *CNGB1* gene is located between the two segregating SNPs (BICF2P309315, BICF2P75954).

We first studied the *retinitis pigmentosa GTPase regulator interacting protein 1* (*RPGRIP1*), originally associated with PRA in the Miniature Longhaired Dachshunds as a candidate gene in our study cohort using the described PCR conditions for the mutation [11]. We found a high carrier frequency in the breed 17.1 % (25/146), but only one affected dog was homozygous for *RPGRIP1* mutation. This leaves the role of *RPGRIP1* unclear and we, therefore, initiated a genome-wide association and linkage analyses to map the PRA locus in a cohort of 6 PRA affected and 14 unaffected dogs. Control dogs were ophthalmologically normal when examined at greater than seven years of age. Statistical analysis by PLINK [20] identified the most highly associated SNP, BICF2P309315 in CFA2 at 61428709 bp (p_raw_ = 4.7×10^−6^, p_genome_ = 0.1) (build 2.1 of the canine genome reference sequence) (Fig. 2A). The association was supported by six closely positioned markers. Another tentative region with two separate SNPs positioned from 70352612 bp to 77628107 bp was found in CFA1 with the best associated SNP, BICF2P839236 (p_raw_ = 7.0×10^−6^, p_genome_ = 0.2). Re-analysis of the data by adjusting for the strongest SNP (BICF2P309315) on CFA2 did not support the association on CFA1 (BICF2P839236, p = 0.9) confirming the association on CFA2. In addition, genotyping of the best associated SNP on CFA1 in a larger cohort of 6 cases and 168 controls (p = 6.97×10^−5^) did not improve the original tentative GWAS association (p = 10^−6^) and strongly suggest that CFA1 does not play a role in the disease etiology. No significant population structure was identified by genome wide IBS clustering (genomic inflation factor  = 1).

The association results were confirmed by a joint family-based linkage and association analysis using a Pseudomarker program. The joint analysis identified the most highly linked region covering nine SNPs at the same locus on CFA2 between 61198715 bp to 64929333 bp, (linkage p = 1.0×10^−6^, association p = 8.7×10^−6^ and joint analysis p = 1.9×10^−7^) (Fig. 2B). A weaker association was identified also in CFA1 (linkage p = 1.0×10^−6^, association p = 2.0×10^−6^ and joint analysis p = 2.0×10^−6^) (data not shown).

Assessment of genotypes at the associated CFA2 locus revealed a shared 1.9 Mb homozygous haplotype block in all of the affected dogs (Fig. 2C), with recessively segregating markers between 61420765-61645929 bp. The associated region contains 37 genes, including a known human RP gene, *CNGB1*.

**Figure 3:**
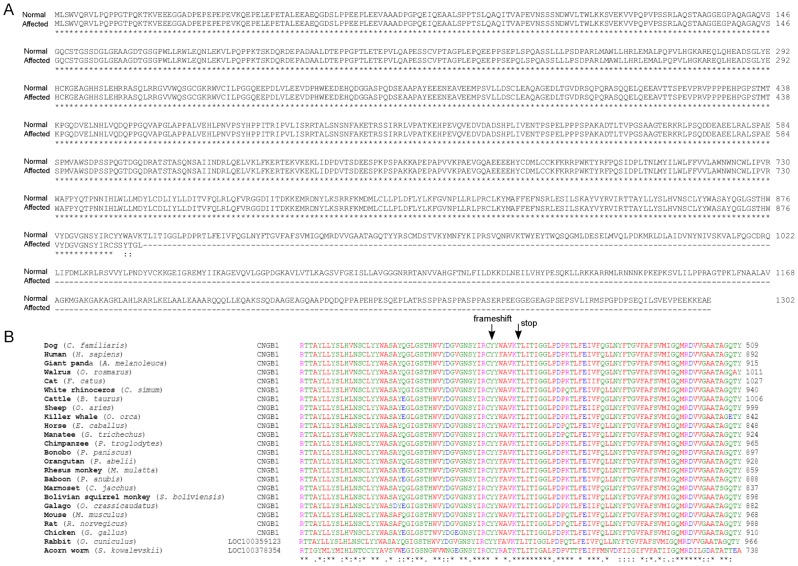
CNGB1 protein alignments. **A)** CNGB1 amino acid alignment of the normal and affected dogs. The p.Tyr889Serfs*5 mutation in the affected dog results in a loss of a significant part of the C-terminus of the protein and probable NMD of the *CNGB1* mRNA **B**) CNGB1 sequence alignment between different vertebrates. The mutation is located in a highly conserved region across species. The arrows mark the first mutated amino acid caused by the frameshift and the premature stop codon.

Based on the exome sequencing data, on average 97 % of the generated raw reads were aligned to CanFam2.1 reference sequence per individual. Of these reads 76 % on average, were aligned to the target region. Each individual had a mean depth of 95 reads on average in the targeted region and 98.9 % of the targeted region was covered with at least one read and about 74 % of the target regions were covered with at least 20 reads (Table S2). The variant calling algorithms detected altogether 129590 single nucleotide variants (SNVs) and 25905 indels across the three individuals. After analyzing the trio according to a recessive mode of inheritance (an affected proband and healthy parent dogs), 204 SNVs and 9 indels were identified. Of the coding variants, two were located in the associated region on CFA2: a 1-bp deletion followed by a 6-bp insertion in the *CNGB1* gene (c.2685delA2687_2688insTAGCTA) (Fig. 2D) and a non-synonymous c.1128G>A, p.376M>I mutation in the *OGFOD1* gene. The indel variant in *CNGB1* causes a frameshift and a premature stop-codon p.Tyr889Serfs*5 in an evolutionary conserved region (Fig. 3A and B), while the missense mutation in *OGFOD1* was predicted to be benign based on the bioinformatics prediction using Polyphen2 [29] and SIFT-programs [30]. We further analyzed the exome data for the region on CFA1, but did not have any case-specific variants, supporting the CFA2 as the causative locus and *CNGB1* mutation as causative for the PRA in the breeds.

To gather further evidence for the *CNGB1* mutation, we genotyped all six PRA affected dogs and all were homozygous indicating a full segregation with the disease. We also genotyped four obligatory carriers from the pedigree (Fig. 1) and they were all mutation carriers. We then genotyped additional 145 randomly selected unaffected Papillons and Phalènes and found four additional dogs homozygous for the mutation and a 17.2% carrier frequency (25/145) was indicated. Of the four homozygous dogs found in the population screening, one was reported to have vision problems in the dim light, which may indicate that the dog is indeed affected with PRA. However, the dog has not had ophthalmologic examination. This dog’s full sibling was also homozygous but neither has been ophthalmologically examined. Two other homozygous dogs had been ophthalmologically examined at very young age (1-2 years), well before the average age of onset of the PRA in the breed and it is likely that the clinical signs are not present at this age. The clinical follow up of these dogs would be important in coming years. We found also 25 carriers of which 52 % have been ophthalmologically examined after 4 years of age and none of them have been diagnosed with PRA. These results indicate a highly significant association between the mutation and disease (P_Fisher_ = 1.4×10^−8^) when comparing genotyped homozygous affected dogs (n = 6) and dogs free of PRA using Fisher’s exact test. The healthy dogs included 4 dogs homozygous for the mutation, 25 heterozygous and 116 dog homozygous for the reference allele.

To gain further support for the causality of the *CNGB1* mutation, we screened additional 334 healthy dogs from 10 other breeds but the mutation was not found. In addition, the mutation was studied in 121 dogs from 44 other breeds affected with PRA or retinal degeneration (Table S3), but none of these affected dogs had the *CNGB1* mutation. Collectively, these results indicate that the *CNGB1* mutation is causal and breed-specific in Papillons and Phalènes.

The entire *CNGB1* mRNA was successfully amplified from a retinal sample from a PRA-free Australian Cattle Dog [Genbank Accession KF551910], indicating that the canine *CNGB1* is also transcribed in the retina.

## Discussion

We have performed a series of genetic analyses, including GWAS, linkage and exome sequencing to identify the mutation for PRA in Papillon and Phalène breeds. Although we managed to establish only a small cohort and pedigree of cases and controls, we mapped a 1.9 Mb locus at CFA2, and confirmed it by both association and linkage approaches according to our pedigree analyses with the expected recessive mode of inheritance. The associated region at CFA2 contains 37 genes, including a known human retinitis pigmentosa gene, *CNGB1.* It is clinically and functionally highly relevant candidate gene for PRA in the Papillon and Phalène breeds. Importantly, our exome data, covering all the coding regions of the associated locus, revealed *CNGB1* to be the only gene with a disease segregating pathogenic mutation: a complex indel variant was found in exon 25 resulting in a premature stop codon and predicting nonsense mediated decay (NMD) of the *CNGB1* mRNA. NMD eliminates the production of mRNA that contains premature translation termination codon and code for nonfunctional polypeptide. In mammalian cells nonsense codons are recognized after splicing and mRNA is programmed to NMD, if there is at least one splicing generated exon-exon junction >50-55 nucleotides downstream of the premature termination codon [31], [32]. As the identified mutation in the *CNGB1* is on exon 25 and the c.2685delA2687_2688insTAGCTA variation is 127 nucleotides from the nearest exon-exon junction downstream, it is hypothesized that the mRNA is subjected to NMD and no protein is translated.

The *CNGB1* gene encodes for a rod photoreceptor cyclic guanosine monophosphate-gated (cGMP) channel β-subunit and is a part of the rod photoreceptor cGMP-gated cation channel complex [33]. This complex consists α and β-subunits and form tetrameric cyclic nucleotide gated (CNG) channels [34], [35]. The visual transduction cascade is mediated by this non-selective cation channel, which is directly gated by cGMP [36]. The channel controls for the flow of sodium and calcium ions through the plasma membrane in response to light induced changes in cGMP level [33]. The β-subunit contains a channel-like structure, but does not form functional subunit without the co-expression of the α-subunit [34], [33]. The α-subunit contains six membrane segments, a voltage sensor region, a pore region and a cGMP-binding motif [37].

The human *CNGB1* comprises of 33 exons and several different transcripts have been found in different tissues [38]. In the retina, at least seven different transcripts are expressed [38]. Rod cells contain a long isoform of the CNGB1 (CNGB1a) [39]. The shorter isoform (CNGB1b) is expressed in the olfactory CNG channels [40] as well as sperm cells and other tissues [41]. The N-terminal part of CNGB1 between exons 1-16 is a glutamic acid-rich and it is also expressed as a separate protein (GARP) [38]. GARP is highly enriched in rod photoreceptors [38]. The C-terminal part is homologous to the α-subunit and forms the channel-like portion. Exons 21-26 are homologous to the transmembrane and pore domains present in the α-subunit, exons 29-31 encodes for the cyclic nucleotide binding domain (CNBD), and exons 19 and 32 codes for domain that are important for the calcium calmodulin/regulation of cGMP affinity [38].

The canine *CNGB1* is similar to human and contains 32 predicted exons based on mRNA (XM_848817.1) sequence alignment. The frameshift mutation in exon 25 predicts that the *CNGB1* mRNA is destroyed by NMD. Therefore, although we lack functional data due to limited access to retinal tissues from the affected dogs, it is likely that the mutation abrogates CNGB1 functions and causes retinal degeneration in Papillons and Phalènes. These breeds provide a model to study the genotype-phenotype correlations related to the possible alternative transcripts and their potential tissue-specific functions.

The defective *CNGB1* has been implicated as a cause of retinal degeneration in human and mouse. Bareil et al. identified a p.G993V missense mutation is a consanguineous French family affected by a night blindness and progressive loss of peripheral vision and ERG recorded rod responses [42]. In another study, a splice site point mutation (c.3444+1G>A) was found in a patient with night blindness, retinal pigment lesion and non-recordable dark adapted flash ERG [43]. In addition, a missense mutation (c.2957A→T; p.N986I) was found in a patient with typical RP with attenuation of the retinal vessels, retinal pigment atrophy and optic disc pallor [44].

In the *CNGB1* ^−/−^ mice, the *CNGB1* gene was found to be important for the formation and outer segment targeting of the rod CNG channels. The *CNGB1*-deficient mice developed retinal degeneration that resembles human RP, progressing from the rod degeneration to a complete loss of rod function and secondary degeneration of the cones [45]. However, the disease in *CNGB1* ^−/−^ mice progresses more slowly when compared to other RP mouse models and the exact events that lead to rod cell death are unclear [45]. In CNGB1^−/−^ mice, 80−90% loss of the rod responses is not detected until at the age 1 year [46]. In human patients, night blindness is usually reported at school age but RP is commonly diagnosed around 30 years of age [42], [43] The PRA in Papillon and Phaléne breeds is diagnosed at the middle age dogs and progresses similar to human and mice.

In summary, we have discovered a putative genetic cause of PRA in the Phalène and Papillon breeds by identifying a frameshift mutation in *CNGB1*. As a further confirmation, the same mutation was also recently reported in an independent study by Petersen-Jones et al. (2013) at the Association for Research in Vision and Ophthalmology (ARVO) conference (unpublished data). Our study enables the development of a genetic test to eradicate the disease from the breeds. The breeds have a high carrier frequency (17.2 %) and careful breeding plans by mating carriers to normal should be considered to maintain genetic diversity. Importantly, our study establishes a large functional knockout animal model for potential therapeutic trials such as gene therapy, and provides a relevant model for further studies of the *CNGB1* biology in the retina and other tissues.

## Supporting Information

Table S1 Primers used for PCR amplification and sequencing of canine CNGB1 mRNA.
(XLSX)

Table S2 Summary of the exome sequencing data analysis of each sequenced individual dog.
(XLSX)

Table S3 Dogs affected with PRA (44 breeds, total 121) were sequenced for the *CNGB1* mutation.
(XLSX)
